# BOOME: A Python package for handling misclassified disease and ultrahigh-dimensional error-prone gene expression data

**DOI:** 10.1371/journal.pone.0276664

**Published:** 2022-10-27

**Authors:** Li-Pang Chen

**Affiliations:** Department of Statistics, National Chengchi University, Taipei, Taiwan, ROC; Utrecht University: Universiteit Utrecht, NETHERLANDS

## Abstract

In gene expression data analysis framework, ultrahigh dimensionality and measurement error are ubiquitous features. Therefore, it is crucial to correct measurement error effects and make variable selection when fitting a regression model. In this paper, we introduce a python package BOOME, which refers to **BOO**sting algorithm for **M**easurement **E**rror in binary responses and ultrahigh-dimensional predictors. We primarily focus on logistic regression and probit models with responses, predictors, or both contaminated with measurement error. The BOOME aims to address measurement error effects, and employ boosting procedure to make variable selection and estimation.

## 1 Introduction

### 1.1 Motivation

Analysis of gene expression data is a popular topic and deserves careful research development. A motivating example in this paper is a gene expression microarray data collected by [1] and explored in some references (e.g., [2]). The full dataset can be found in the R package SIS. The data contain binary responses, acute myeloid leukemia (AML) and acute lymphoblastic leukemia (ALL), and 7128 gene expression levels that were measured using Affymetrix oligonucleotide arrays. In addition, samples with size 72 come from the two classes, with 47 specimens in class ALL and 25 specimens in class AML. The primary objective of this study is to characterize the relationship between leukemia and gene expression values, and see how gene expression values interpret leukemia. To achieve this goal, a commonly used approach is to build a regression model by treating leukemia and gene expression values as binary responses and the predictors, respectively. To model a binary response, logistic regression or probit models are perhaps frequently implemented parametric approaches.

According to this gene expression data, ultrahigh dimensionality (*p* ≫ *n*) is a challenging feature. Since not every gene expression value is informative, using irrelevant predictors in regression models may affect the performance of classification and induce wrong conclusions. Therefore, making variable selection and retaining important ones are needed. While variable selection techniques have been widely explored (e.g., [3–5]), those strategies may fail to handle the case that the dimension is extremely larger than the sample size. The other concern is measurement error in the response and predictors. As discussed in [6–8], gene expression values may be measured imprecisely due to unadjusted machines. Moreover, as commented by [9], it is also possible to falsely record ALL (or AML) to AML (or ALL), known as misclassification, because the microscopy images of AML bone marrow cells contain many immature granulocytes and monocytes, and ALL bone marrow cell microscopy images contain many immature lymphocytes. It is known that ignoring measurement error effects may cause tremendous biases and induce incorrect decisions, such as the false exclusion of truly informative predictors or false inclusion of irrelevant predictors when making variable selection (e.g., [6, 10, 11]). Therefore, it is crucial to correct measurement error effects. In particular, unlike existing literature that handles either variable selection or measurement error, the main challenge of this dataset is to correct measurement error and select informative predictors under ultrahigh-dimensional data *simultaneously*, and measurement error may explicitly affect the performance of variable selection. In other words, truly noninformative predictors may be falsely included if measurement error effects are ignored (e.g., [6, 11, 12]). As a result, it is necessary to suitably adjust measurement error effects and then use the corrected version to make variable selection.

### 1.2 Contributions

To address those concerns, we develop a package BOOME that is now available on https://pypi.org/project/BOOME/0.0.2/. The purpose of this package is to correct two measurement error processes in responses and predictors, and employ the boosting procedure to retain important predictors and estimate nonzero coefficients simultaneously.

In standard analysis of regression models, to estimate unknown parameters, one may require to derive likelihood functions, or more generally, unbiased estimating functions. Then the resulting estimator can be obtained by optimizing the constructed estimating functions. In the presence of measurement error, however, naively adopting error-prone predictors to the estimating functions would yield the biased estimators (e.g., [10, 13]). Therefore, to address this challenge, as discussed in measurement error framework, one should derive the corrected estimating functions with measurement error effects eliminated before implementing computational algorithms or estimation methods to derive the estimator, which is the standard step in measurement error analysis (e.g., [6, 10–18]). Following this idea, our strategy is to derive a new estimating function with measurement error effects in responses and predictors corrected, then adopt it to select informative predictors and obtain the corresponding estimators. Specifically, to correct measurement error effects to the binary response, we define the misclassification matrix (e.g., [13], p.131), which is formulated by specificity and sensitivity (e.g., [13], p.70), and will be described in details in Section 2.2, to adjust for measurement error effects in the responses and derive a new corrected response. Regarding the error-prone predictors, we adopt the sufficient statistics of the predictors and the regression calibration to correct measurement error effects to the predictors. Based on such strategies of measurement error corrections, we develop the corrected estimating functions under logistic regression or probit models, respectively. After that, we implement the corrected estimating functions to the boosting algorithm to make variable selection and estimation (e.g., [19]; [20], p.608). Detailed descriptions of measurement error corrections and the boosting algorithm are deferred to Sections 3.1 and 3.2, respectively.

### 1.3 Comparisons

Variable selection and estimation with correction of measurement error have been discussed, and many methods based on different settings have been developed. For example, [21] consider generalized linear models (GLM) and proposed the generalized matrix uncertainty selector (GMUS), whose idea is based on a Taylor series expansion of the GLM mean function around the true, but unknown, predictors. [22] considered parametric and semi-parametric regression models with error-prone predictors, and developed a corrected estimating equation to make variable selection. For survival data with incomplete responses, [6, 11, 12] considered several types of survival models and developed penalized estimating function to deal with variable selection. [23] proposed the MEBoost method, which adopts the boosting method to select informative variables under error-prone linear regression models. While many methods have been developed, they primarily focus on measurement error in predictors and rare work has been available to address measurement error effects in responses. In addition, although the boosting method has been applied to error-prone data, the existing method simply focuses on linear regression models, and other types of regression models have not been explored.

In the past developments, several existing packages based on different software have been developed to deal with either measurement error or variable selection. To name a few, for the R software, two packages glmnet [24] and SIS [25] are popular methods to handle variable selection. For the Python software, xverse [26] can be adopted to do feature selection. However, they fail to deal with measurement error effects. On the other hand, the two packages GLSME [27] and mecor [28] in the R software focus on linear models and aim to adjust for measurement error effects in the response and/or predictors, but they cannot deal with variable selection.

Compared with existing packages, there are some differences from the package BOOME. Specifically, our package is able to handle ultrahigh-dimensionality and mismeasured data simultaneously. Unlike most existing frameworks that focus on measurement error in predictors or continuous responses, our approach extends measurement error in binary responses (a.k.a misclassification), and the model structure for misclassification is more complex than that under continuous responses. Our approach can deal with error-prone response and predictors simultaneously. Moreover, boosting iteration may reduce the possibility of falsely excluding important predictors and enhance the accuracy of the estimator. Most importantly, our development is based on the Python language, and, to the best of knowledge, there is no relevant development in Python packages.

### 1.4 Organization of this paper

The remainder is organized as follows. In Section 2, we introduce two regression models to characterize binary responses. In addition, we introduce two measurement error models to describe error-prone responses and predictors, respectively. In Section 3, we present the BOOME method. Specifically, we first discuss some valid strategies to handle measurement error effects, and then discuss the boosting method for variable selection and estimation. In Section 4, we introduce the Python package BOOME, including some important functions as well as their implementation. In Section 5, we demonstrate the application of the package BOOME and analyze the gene expression data. Moreover, we also demonstrate simulation studies. Finally, a general discussion is presented in Section 6.

## 2 Regression models

### 2.1 Regression models with binary responses

Following the motivating example in Section 1.1, let *n* = 72 denote the sample size. For *i* = 1, …, *n*, let *Y*_*i*_ be a binary response where *Y*_*i*_ = 1 represents AML and *Y*_*i*_ = 0 indicates ALL. Moreover, let *X*_*i*_ be a *p*-dimensional vector of gene expressions with *p* = 7128.

With the absence of measurement error effects, our goal is to use the gene expression values *X*_*i*_ to characterize the disease *Y*_*i*_ through a *p*-dimensional vector of parameters *β*. In the framework of GLM, logistic regression or probit models are commonly used. Specifically, the logistic regression model (LR) is formulated by

$$\begin{array}{c} P(Y_{i}=1|X_{i})=\frac{\text{exp}(X_{i}^{⊤}\beta )}{1+\text{exp}(X_{i}^{⊤}\beta )}; \end{array}$$ (1)

and the probit model (PM) is given by

$$\begin{array}{c} P(Y_{i}=1|X_{i})=\Phi \left(X_{i}^{⊤}\beta \right), \end{array}$$ (2)

where Φ(⋅) is the cumulative distribution function of the standard normal distribution.

To estimate *β*, a common strategy is to optimize the likelihood function, or equivalently, solve the estimating equation. Specifically, for *i* = 1, …, *n*, the estimating function based on (1) is defined as

$$\begin{array}{c} g_{\text{LR}}\left(\beta \right)=-\frac{1}{n}\sum_{i=1}^{n}\{X_{i}Y_{i}-\frac{X_{i}\text{exp}\left(X_{i}^{⊤}\beta \right)}{1+\text{exp}(X_{i}^{⊤}\beta )}\}; \end{array}$$ (3)

and the estimating function based on (2) is given by

$$\begin{array}{c} g_{\text{PM}}\left(\beta \right)=-\frac{1}{n}\sum_{i=1}^{n}[\frac{X_{i}}{\sqrt{2\pi }}\text{exp}\{-\frac{1}{2}{\left(X_{i}^{⊤}\beta \right)}^{2}\}\{\frac{Y_{i}}{\Phi (X_{i}^{⊤}\beta )}+\frac{Y_{i}-1}{1-\Phi (X_{i}^{⊤}\beta )}\}]. \end{array}$$ (4)

Solving *g*_LR_(*β*) = 0 or *g*_PM_(*β*) = 0 yields the estimator of *β*.

### 2.2 Measurement error models

In applications, as discussed in Section 1.1, *Y*_*i*_ and *X*_*i*_ might be subject to measurement error due to wrong records by investigators or imprecise measurements by unadjusted machines. Under this scenario, we particularly denote *Y*_*i*_ and *X*_*i*_ as unobserved variables, and let $Y_{i}^{*}$ and $X_{i}^{*}$ denote the surrogate measurements of *Y*_*i*_ and *X*_*i*_, respectively, and they are recorded in the data.

We now provide an intuition of modeling error-prone data. Let *f*(⋅|⋅) represent the conditional distribution for variables indicated by the corresponding arguments, and let *f*(⋅) denote marginal or joint distribution of random variables. Following the similar discussion in [13] (Chapter 8), we consider the joint distribution $f(Y_{i},Y_{i}^{*},X_{i},X_{i}^{*})$ and factorize it as

$$\begin{array}{ccc} f(Y_{i},Y_{i}^{*},X_{i},X_{i}^{*}) & = & f\left(Y_{i}^{*}|Y_{i},X_{i}^{*},X_{i}\right)\times f\left(Y_{i}|X_{i}^{*},X_{i}\right)\times f\left(X_{i}^{*}|X_{i}\right)\times f\left(X_{i}\right)\\ & = & f\left(Y_{i}^{*}|Y_{i},X_{i}\right)\times f\left(Y_{i}|X_{i}\right)\times f\left(X_{i}^{*}|X_{i}\right)\times f\left(X_{i}\right), \end{array}$$ (5)

where the second step is obtained by the nondifferential $X_{i}^{*}$. With the marginal distribution *f*(*X*_*i*_) left specified, the factorization (5) says that the inference about *f*(*Y*_*i*_|*X*_*i*_) is conducted based on examining $f(Y_{i}|X_{i}^{*},X_{i})$ with the predictor measurement error process being characterized by $f(X_{i}^{*}|X_{i})$, and $f(Y_{i}^{*}|Y_{i},X_{i})$ facilitates the response measurement error process.

To analyze measurement error effects when constructing regression models, we first need to characterize the relationship between *Y*_*i*_ and $Y_{i}^{*}$ as well as *X*_*i*_ and $X_{i}^{*}$. Specifically, to connect *Y*_*i*_ and $Y_{i}^{*}$, we consider the conditional probability

$$\begin{array}{c} \pi_{ikl}=P\left(Y_{i}^{*}=k|Y_{i}=l,X_{i}\right) \end{array}$$ (6)

for *k*, *l* ∈ {0, 1}, satisfying *π*_*i*10_+ *π*_*i*00_ = 1 and *π*_*i*01_+ *π*_*i*11_ = 1, where *π*_*i*11_ and *π*_*i*00_ are called *sensitivity* and *specificity*, respectively, or known as classification probabilities; *π*_*i*10_ and *π*_*i*01_ are known as misclassification probabilities (e.g., [13], p.70). Moreover, to characterize *π*_*i*01_ and *π*_*i*10_, logistic regression (1) or probit models (2) with additional parameter *γ* are suitable choices. By the law of total probability, $P(Y_{i}^{*}=1|X_{i})$ and $P(Y_{i}^{*}=0|X_{i})$ can be expressed as

$$\begin{array}{c} (\begin{array}{c} P(Y_{i}^{*}=1|X_{i})\\ P(Y_{i}^{*}=0|X_{i}) \end{array})=\Pi_{i}(\begin{array}{c} P(Y_{i}=1|X_{i})\\ P(Y_{i}=0|X_{i}) \end{array}), \end{array}$$ (7)

where $\Pi_{i}=\left(\begin{array}{cc} \pi_{i11} & \pi_{i10}\\ \pi_{i01} & \pi_{i00} \end{array}\right)$ is called a 2 × 2 misclassification matrix (e.g., [13], p.131) that is assumed to be invertible.

Next, to describe the relationship between $X_{i}^{*}$ and *X*_*i*_, we employ the classical measurement error model

$$\begin{array}{c} X_{i}^{*}=X_{i}+\epsilon_{i}, \end{array}$$ (8)

where *ϵ*_*i*_ is independently and identically distributed normal distribution *N*(0, Σ_*ϵ*_) with Σ_*ϵ*_ being a *p* × *p* covariance matrix representing the magnitude of measurement error effects in the predictors. We assume that *ϵ*_*i*_ is independent of *X*_*i*_.

## 3 Method

### 3.1 Correction of measurement error

In this section, we primarily correct measurement error effects to responses and predictors.

Motivated by (7), by multiplying the inverse matrix of Π_*i*_ to both sides of (7), we can obtain that

$$\begin{array}{c} P\left(Y_{i}=1|X_{i}\right)=\frac{P\left(Y_{i}^{*}=1|X_{i}\right)-\pi_{i10}}{1-\pi_{i10}-\pi_{i01}}, \end{array}$$ (9)

which indicates that the unobserved response *Y*_*i*_ = 1 can be implicitly characterized by $Y_{i}^{*}=1$ with the adjustment in terms of *π*_*i*01_ and *π*_*i*10_. It motivates us to consider the “corrected” response, denoted $Y_{i}^{**}$, which satisfies

$$\begin{array}{c} P\left(Y_{i}^{**}=1|X_{i}\right)=\frac{P\left(Y_{i}^{*}=1|X_{i}\right)-\pi_{i10}}{1-\pi_{i10}-\pi_{i01}}, \end{array}$$ (10)

suggesting that

$$\begin{array}{c} Y_{i}^{**}=\frac{Y_{i}^{*}-\pi_{i10}}{1-\pi_{i10}-\pi_{i01}}. \end{array}$$ (11)

In addition, (11) indicates that $E(Y_{i}^{**}|Y_{i},X_{i})=Y_{i}$, verifying that (11) is a suitable correction to recover *Y*_*i*_. Moreover, we note that (11) holds regardless of the choice of regression models because it is obtained by the equalities (9) and (10) where the conditional probability can be (1) or (2).

To correct measurement error effects to the predictors, we provide two different strategies for different models. For the logistic regression model in terms of $Y_{i}^{**}$ and unobserved *X*_*i*_, we follow the similar discussion in [18] and aim to replace *X*_*i*_ by its sufficient statistic:

$$\begin{array}{c} X_{\text{SS},i}^{**}=X_{i}^{*}+Y_{i}^{**}\Sigma_{\epsilon }\beta , \end{array}$$ (12)

which can be regarded as correction of $X_{i}^{*}$. Replacing *Y*_*i*_ and *X*_*i*_ in (1) by (11) and (12) gives the corrected estimating function:

$$\begin{array}{ccc} g_{\text{LR}}^{**}\left(\beta \right) & = & -\frac{1}{n}\sum_{i=1}^{n}[\left(X_{i}^{*}+Y_{i}^{**}\Sigma_{\epsilon }\beta \right)Y_{i}^{**}\\ & & -\left(X_{i}^{*}+Y_{i}^{**}\Sigma_{\epsilon }\beta \right)\frac{\text{exp}\{{\left(X_{i}^{*}+Y_{i}^{**}\Sigma_{\epsilon }\beta \right)}^{⊤}\beta \}}{1+\text{exp}\{{\left(X_{i}^{*}+Y_{i}^{**}\Sigma_{\epsilon }\beta \right)}^{⊤}\beta \}}]. \end{array}$$ (13)

On the other hand, to handle measurement error effects to the probit model, we adopt the regression calibration (e.g., [10], Chapter 4), whose key idea is to replace *X*_*i*_ by the conditional expectation $E(X_{i}|X_{i}^{*})$. By the best linear unbiased prediction, it can be expressed as (e.g., [11, 15])

$$\begin{array}{c} E\left(X_{i}|X_{i}^{*}\right)=\mu_{X}+{\left(\Sigma_{X^{*}}-\Sigma_{\epsilon }\right)}^{⊤}\Sigma_{X^{*}}^{-1}\left(X_{i}^{*}-\mu_{X^{*}}\right), \end{array}$$

where *μ*_*X*_ and *μ*_X*_ represent the mean vectors of *X* and X*, respectively, and Σ_X*_ represents the covariance matrix of X*. Since *μ*_*X*_ = *μ*_X*_, by the method of moments, we obtain that

$$\begin{array}{c} X_{i}^{**}≜{\hat{\mu }}_{X^{*}}+{\left({\hat{\Sigma }}_{X^{*}}-\Sigma_{\epsilon }\right)}^{⊤}{\hat{\Sigma }}_{X^{*}}^{-1}\left(X_{i}^{*}-{\hat{\mu }}_{X^{*}}\right), \end{array}$$ (14)

where ${\hat{\mu }}_{X^{*}}$ and ${\hat{\Sigma }}_{X^{*}}$ are empirical estimates of *μ*_X*_ and Σ_X*_, respectively. Consequently, replacing *Y*_*i*_ and *X*_*i*_ in (2) by (11) and (14) gives the corrected estimating function:

$$\begin{array}{c} g_{\text{PM}}^{**}\left(\beta \right)=-\frac{1}{n}\sum_{i=1}^{n}[\frac{X_{i}^{**}}{\sqrt{2\pi }}\text{exp}\{-\frac{1}{2}{\left(X_{i}^{**⊤}\beta \right)}^{2}\}\{\frac{Y_{i}^{**}}{\Phi (X_{i}^{**⊤}\beta )}+\frac{Y_{i}^{**}-1}{1-\Phi (X_{i}^{**⊤}\beta )}\}]. \end{array}$$ (15)

### 3.2 Boosting algorithm

Let g**(*β*) denote the unified notation to represent the corrected estimating function (13) or (15). To make variable selection and estimation for *β*, we adopt the boosting algorithm with the correction of measurement error effects. The proposed method is called BOOME, and the procedure is summarized in Algorithm 1.

Specifically, the algorithm starts by an initial value *β*^(0)^ taken by the *p*-dimensional zero vector 0_*p*_. Suppose that we run *T* times iterations, and for each iteration step *t* = 1, …, *T*, we compute the estimating function g**(*β*) evaluated at the (*t* − 1)th iterated value *β*^(*t*−1)^, and denote it as Δ^(*t*−1)^. After that, we define the active set $J_{t-1}$ that collects the indexes satisfying $\left|\Delta_{j}^{\left(t-1\right)}\right|\geq \tau \;\underset{j^{\prime }}{\text{max}}\left|\Delta_{j^{\prime }}^{\left(t-1\right)}\right|$, where *τ* ∈ [0, 1] is a constant and $\Delta_{j}^{\left(t-1\right)}$ is the *j*th component in a vector Δ^(*t*−1)^. It implies that the active set $J_{t-1}$ aims to retain informative predictors by treating $\Delta_{j}^{\left(t-1\right)}$ as a signal. Finally, for those $j\in J_{t-1}$, we update the iterated value of the *j*th component in *β*^(*t*−1)^, say $\beta_{j}^{\left(t-1\right)}$, by adding an increment $\eta \cdot \Delta_{j}^{\left(t-1\right)}$ for some positive constant *η*. Repeating those steps *T* times yields the final estimator of *β*.

In Algorithm 1, *τ*, *η*, and *T* can be user-specific and may affect the iteration result. Similar to the comment in [29], the algorithm satisfying *Tη* → 0 as *T* → ∞ and *η* → 0 is approximately equivalent to the LASSO method. Therefore, it suggests taking *η* as a small value, such as *η* = 0.01, in applications. On the other hand, while *T* is suggested being large, it may cause over-fitting. To provide a suitable *T* and stop the iteration earlier, we suggest a criterion: the iteration stops at *T* if

$$\begin{array}{c} ∥g^{**}\left(\beta^{\left(T\right)}\right)-g^{**}\left(\beta^{\left(T+1\right)}\right)∥<\xi \end{array}$$

is satisfied for some positive constant *ξ*. Finally, for the choice of *τ*, one may adopt some criteria such as cross-validation (e.g., [19]).

**Algorithm 1:** Boosting Procedure in BOOME

Let *β*^(0)^ = 0_*p*_ denote an initial value;

**for** *step t with t* = 1, 2, …, *T* **do**

 (a) calculate Δ^(*t*−1)^ = g**(*β*)|_*β* = *β*^(*t*−1)^_;

 (b) determine $J_{t-1}=\{j:\left|\Delta_{j}^{\left(t-1\right)}\right|\geq \tau \;\underset{j}{\text{max}}\left|\Delta_{j}^{\left(t-1\right)}\right|\}$;

 (c) update $\beta_{j}^{\left(t\right)}\leftarrow \beta_{j}^{\left(t-1\right)}+\eta \cdot \Delta_{j}^{\left(t-1\right)}$ for all $j\in J_{t-1}$, and define $\beta^{\left(t\right)}=(\beta_{1}^{\left(t\right)},\ldots ,\beta_{p}^{\left(t\right)})$;

The final estimator is given by $\hat{\beta }≜\beta^{\left(T\right)}$.

## 4 Description and implementation of BOOME

We develop a Python package, called BOOME, to implement the variable selection and estimation with measurement error correction described in Section 3. The package BOOME contains three functions: ME_Generate, LR_Boost, and PM_Boost. The function ME_Generate aims to generate artificial data under specific models listed in Section 2.1 and error-prone predictors. The functions LR_Boost and PM_Boost implement the boosting procedure in Algorithm 1, except for the difference that LR_Boost is based on the logistic regression model, and PM_Boost focuses on the probit model. We now describe the details of these three functions.

### 4.1 ME_Generate

We use the following command to obtain the artificial data:

ME_Generate(n,beta,matrix,X,gamma)

where the meaning of each argument is described as follows:

- n: The number of observations.
- beta: A *p*-dimensional vector of parameter *β* specified by users.
- matrix: A user-specific covariance matrix implemented to (8).
- X: A user-specific *n* × *p* matrix of predictors.
- gamma: A *p*-dimensional vector of parameter *γ* in *π*_*i*10_ and *π*_*i*01_ specified by users.

The function ME_Generate returns a list of components:

- data: A dataset with error-prone predictors and responses. It is a *n*×(*p*+ 1) data frame, where the column with label y represents the error-prone response, and the column with label *j*, *j* = 1, …, *p*, represents the *j*th error-prone predictor $X_{j}^{*}$.
- pr: Two misclassification probabilities *π*_*i*10_ and *π*_*i*01_ in (7).

### 4.2 LR_Boost

To demonstrate Algorithm 1 with the corrected estimating function (13) for the logistic regression model, we adopt the following command:

LR_Boost(X,Y,ite,thres,correct_X,correct_Y,pr,lr,matrix)

where the meaning of each argument is described as follows:

- X: A *n* × *p* matrix of continuous predictors that are precisely measured or subject to measurement error.
- Y: A *n*-dimensional vector of binary responses that are precisely measured or subject to measurement error.
- ite: A number of iteration *T* in Algorithm 1.
- thres: A threshold value *τ* in Algorithm 1.
- correct_X: Determine the correction of measurement error in predictors. Select “1” if correction is needed, and “0” otherwise.
- correct_Y: Determine the correction of measurement error in the response. Select “1” if correction is needed, and “0” otherwise.
- pr: Two misclassification probabilities *π*_*i*10_ and *π*_*i*01_ in (7).
- lr: A learning rate *η* in Algorithm 1.
- matrix: A *p* × *p* covariance matrix Σ_*ϵ*_ in (8).

The function LR_Boost returns a list of components:

- estimated coefficients: the *p*-dimensional vector of estimators of *β*.
- predictors: Indexes of nonzero values in estimated coefficients.
- number of predictors: The number of nonzero values in estimated coefficients.

### 4.3 PM_Boost

To make variable selection and estimation for probit model by using Algorithm 1 with the corrected estimating function (15), we implement the following function:

PM_Boost(X,Y,ite,thres,correct_X,correct_Y,pr,lr,matrix)

The arguments in PM_Boost as well as the output produced by PM_Boost are the same as those in LR_Boost.

## 5 Numerical studies

In this section, we implement the functions in the package BOOME to analyze a real dataset as well as demonstrate simulation studies. Detailed code demonstrations are also available on the pypi website https://pypi.org/project/BOOME/0.0.2/.

### 5.1 Analysis of gene expression microarray data

**Fig 1:**
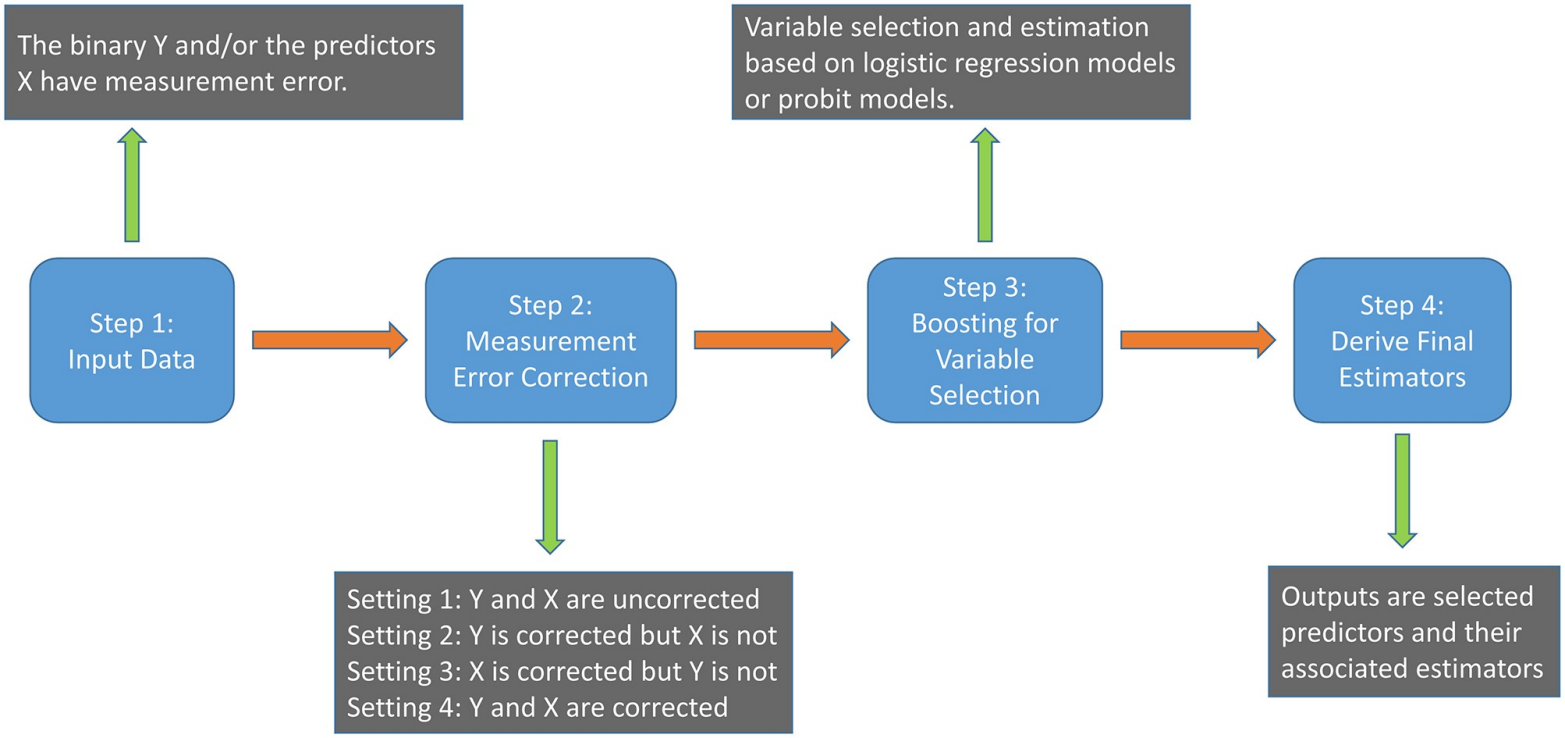
Diagram of data analysis and implementation of the package BOOME.

In this section, we implement the package BOOME to analyze a gene expression microarray data introduced in Section 1.1. The steps for analysis are summarized in Fig 1. As shown in Step 1 of Fig 1, we recognize that, for *i* = 1, …, *n*, $Y_{i}^{*}$ is the binary random variable with outcomes AML and ALL that may possibly subject to misclassification, and $X_{i}^{*}$ represents the gene expression values that are contaminated with measurement error. Before analyzing this dataset, we first standardize all predictors, such that the mean and the variance of each predictor become 0 and 1, respectively. Let data_GE in Python code represent the gene expression microarray data that we introduced in Section 1.1, where the first column is the binary outcome and the remaining columns are gene expression values. Based on this dataset, the following code shows the input of gene expression data and the standardized procedure:

$$\begin{array}{ccc} & & \text{x}=\text{data}\_\text{GE}.\text{drop}\left({[}^{\prime }{\text{y}}^{\prime }],\text{axis}=1\right);\;\;\text{y}=\text{data}\_\text{GE}\left[{[}^{\prime }{\text{y}}^{\prime }]\right]\\ & & \text{zscore}=\text{preprocessing}.\text{StandardScaler}()\\ & & \text{x}\_\text{z}=\text{pd}.\text{DataFrame}(\text{zscore}.\text{fit}\_\text{transform}(\text{x}))\\ & & \text{x}\_\text{z}.\text{columns}=\text{range}(1,7129)\\ & & \text{data}\_\text{z}1=\text{pd}.\text{concat}([\text{x}\_\text{z},\text{y}],\text{axis}=1) \end{array}$$

To examine the impact of measurement error effects, we primarily consider four settings in Step 2 of Fig 1:

- Setting 1: $Y_{i}^{*}$ and $X_{i}^{*}$ are not corrected.
- Setting 2: $Y_{i}^{*}$ is corrected while $X_{i}^{*}$ is not.
- Setting 3: $X_{i}^{*}$ is corrected while $Y_{i}^{*}$ is not.
- Setting 4: $Y_{i}^{*}$ and $X_{i}^{*}$ are corrected.

Here Setting 1 aims to implement Algorithm 1 to the estimating functions (3) or (4) with *Y*_*i*_ and *X*_*i*_ replaced by error-prone variables $Y_{i}^{*}$ and $X_{i}^{*}$, respectively. Setting 2 considers the estimating function in Setting 1 with $Y_{i}^{*}$ replaced by corrected responses (11); and Setting 3 adopts the estimating function in Setting 1 with $X_{i}^{*}$ replaced by corrected predictors (12) or (14). Setting 4 uses Algorithm 1 to estimating functions (13) or (15), where measurement error in both responses and predictors are corrected. As discussed in Section 1.1, both responses and predictors are contaminated with measurement error, then Setting 4 is the proposed method by correcting measurement error effects in responses and predictors. On the other hand, Settings 1-3, known as *naive* methods, reflect that measurement error in leukemia, gene expressions, or both are not corrected. Basically, Settings 1-3 are considered to show the impact of ignorance of measurement error effects and are compared with the proposed method in Setting 4.

In Step 3, we implement the functions in BOOME for four different settings. We first note that the dataset has no additional information, such as repeated measurements or validation data, to estimate parameters Σ_*ϵ*_ in measurement error models as well as two misclassification probabilities *π*_*i*10_ and *π*_*i*01_, here we conduct sensitivity analyses, which specify reasonable values for Σ_*ϵ*_ and enable us to examine the impact of different magnitudes of measurement error. In our study, we specify Σ_*ϵ*_ as a diagonal matrix with diagonal entries $\sigma_{\epsilon }^{2}$ being 0.2, 0.5, and 0.7. For the implementation, we take $\sigma_{\epsilon }^{2}=0.2$ as an example and use the following command to demonstrate Σ_*ϵ*_, denoted as matrix:

$$\begin{array}{c} \text{matrix}=0.2*\text{np}.\text{identity}(7128) \end{array}$$

With $\sigma_{\epsilon }^{2}$ being specified, we further determine misclassification probabilities *π*_*i*10_ and *π*_*i*01_. Specifically, since *π*_*i*10_ and *π*_*i*01_ defined in (6) rely on *X*_*i*_, we reproduce *X*_*i*_ by (8), where $X_{i}^{*}$ is observed gene expression values and *ϵ*_*i*_ is generated from a normal distribution with $\sigma_{\epsilon }^{2}$ given by 0.2, 0.5 or 0.7. After that, we adopt logistic regression or probit models to characterize (6) with the corresponding parameter specified as *γ* ≜ 1_*p*_, where 1_*p*_ is a *p*-dimensional vector with all entries being one. Therefore, values of *π*_*i*10_ and *π*_*i*01_ are obtained. To show the demonstration, we summarize the following function pi that is used to implement this idea and compute *π*_*i*10_ and *π*_*i*01_. The resulting values of *π*_*i*10_ and *π*_*i*01_ are denoted as pr:

$$\begin{array}{ccc} & & \text{def}\;\text{pi}(\text{df},\text{cov}):\\ & & \;\;\;\;\text{x}=\text{np}.\text{array}(\text{df}.\text{drop}\left({[}^{\prime }{\text{y}}^{\prime }],\text{axis}=1\right))\\ & & \;\;\;\;\text{p}=\text{df}.\text{shape}[1]-1\\ & & \;\;\;\;\text{n}=\text{len}(\text{df})\\ & & \;\;\;\;\text{def}\;\text{mean}(\text{p}):\\ & & \;\;\;\;\;\;\;\;\text{zero}=[0]\\ & & \;\;\;\;\;\;\;\;\text{return}\;\text{zero}*\text{p}\\ & & \;\;\;\;\text{p}=\text{df}.\text{shape}[1]-1\\ & & \;\;\;\;\text{m}=[]\\ & & \;\;\;\;\text{p}=\text{df}.\text{shape}[1]-1\\ & & \;\;\;\;\text{for}\;\text{i}\;\text{in}\;\text{range}(\text{n}):\\ & & \;\;\;\;\;\;\;\;\text{p}=\text{df}.\text{shape}[1]-1\\ & & \;\;\;\;\text{e}=\text{np}.\text{array}(\text{np}.\text{random}.\text{multivariate}\_\text{normal}(\text{mean}(\text{p}),\text{cov}))\\ & & \;\;\;\;\;\;\;\;\text{p}=\text{df}.\text{shape}[1]-1\\ & & \;\;\;\;\;\;\;\;\text{x}=\text{np}.\text{array}(\text{df}.\text{T}[\text{i}][:\text{p}])-\text{e}\\ & & \;\;\;\;\;\;\;\;\text{m}\_\text{list}=[]\\ & & \;\;\;\;\;\;\;\;\text{scalar}=\text{math}.\text{exp}(1+\text{np}.\text{dot}(\text{x},\text{np}.\text{array}([1]*\text{p})))\\ & & \;\;\;\;\;\;\;\;\text{matrix}=\text{np}.\text{array}([[1/(1+\text{scalar}),\text{scalar}/(1+\text{scalar})],\\ & & \;\;\;\;\;\;\;\;\;\;\;\;\;\;\;\;[\text{scalar}/(1+\text{scalar}),1/(1+\text{scalar})]])\\ & & \;\;\;\;\;\;\;\;\text{m}\_\text{list}.\text{append}(\text{matrix}[0][1])\\ & & \;\;\;\;\;\;\;\;\text{m}\_\text{list}.\text{append}(\text{matrix}[1][0])\\ & & \;\;\;\;\;\;\;\;\text{m}.\text{append}(\text{m}\_\text{list})\\ & & \;\;\;\;\text{m}=\text{np}.\text{array}(\text{m})\\ & & \;\;\;\;\text{return}\;\text{m}\\ & & \text{pr}=\text{pi}(\text{data}\_\text{z}1,\text{matrix}) \end{array}$$

We now implement two functions LR_Boost and PM_Boost to analyze the data, where, for logistic regression model, *T* is given by 1000, *η* is set as 0.01, and *τ* is equal to 0.9; for probit models, *T* is given by 2000, *η* is set as 0.01, and *τ* is equal to 0.9.

Detailed implementations of the proposed method are described below, keeping in mind that we demonstrate correct_X = 1 and correct_Y = 1 to show the proposed method (Setting 4); different values for arguments correct_X and correct_Y reflect different settings mentioned above.

$$\begin{array}{ccc} & & \text{LR}\_\text{Boost}(\text{X},\text{Y},\;\text{ite}=1000,\;\text{thres}=0.9,\;\text{correct}\_\text{X}=1,\;\text{correct}\_\text{Y}=1,\;\text{pr},\;\\ & & \;\;\;\;\;\;\;\;\text{lr}=0.01,\;\text{matrix})\\ & & \text{PM}\_\text{Boost}(\text{X},\;\text{Y},\;\text{ite}=2000,\;\text{thres}=0.9,\;\text{correct}\_\text{X}=1,\;\text{correct}\_\text{Y}=1,\;\text{pr},\;\\ & & \;\;\;\;\;\;\;\;\text{lr}=0.01,\;\text{matrix}) \end{array}$$

In Step 4 of Fig 1, we report the estimation results. To save limited space and provide precise information, we summarize the predictors and their estimates that are commonly selected under $\sigma_{\epsilon }^{2}=0.2,\;0.5$ or 0.7, and numerical results for all settings obtained by (1) and (2) are placed in Tables 1 and 2, respectively. Moreover, to see the impacts of different regression models, we summarize commonly chosen predictors in Table 3.

**Table 1:** Estimation results based on the logistic regression model.

#	Setting 1		Setting 2		Setting 3		Setting 4	
	ID	EST	ID	EST	ID	EST	ID	EST
1	134	-0.208	7	0.41	134	-0.04	7	0.438
2	412	-0.606	268	-0.684	412	-0.484	268	-0.338
3	760	0.271	731	-1.161	635	-0.186	731	-1.456
4	804	-1.271	746	-0.11	738	0.196	746	-0.045
5	1144	-0.09	1024	-0.032	760	0.271	1089	-0.12
6	1207	-0.108	1089	-0.133	804	-1.301	1148	0.584
7	1239	-0.566	1148	0.475	1144	-0.123	1307	0.446
8	1260	0.786	1307	0.742	1207	-0.115	1588	-0.132
9	1669	0.155	1687	-0.153	1239	-0.234	1687	-0.085
10	1685	-0.169	1708	-0.229	1260	0.912	1858	1.043
11	1834	1.471	1724	0.049	1669	0.013	1987	0.327
12	1882	1.337	1858	1.126	1685	-0.122	2039	1.184
13	1928	-1.229	1987	0.46	1834	1.496	2059	-0.581
14	1997	0.132	2039	0.903	1882	1.271	2065	-0.294
15	2121	0.033	2047	-0.07	1928	-1.221	2377	1.677
16	2288	0.414	2057	-0.175	1997	0.155	2460	0.281
17	2354	-0.087	2059	-0.169	2288	0.858	2469	-0.039
18	2441	-0.032	2208	0.166	2354	-0.085	2534	-0.799
19	2737	0.051	2377	1.389	2737	0.054	2895	0.035
20	2797	0.052	2460	0.55	2797	0.075	2941	0.058
21	3183	0.376	2534	-0.969	3183	0.228	3276	0.104
22	3252	0.464	2682	0.169	3252	0.801	3432	-0.631
23	3714	0.078	2895	0.102	3714	0.022	3660	0.07
24	3847	1.529	2941	0.733	3847	1.553	3771	-1.121
25	4211	-0.578	3276	0.112	4030	-0.01	3810	-0.298
26	4291	-0.66	3432	-0.378	4211	-0.367	3997	-0.069
27	4328	-0.082	3478	-0.029	4291	-0.88	4054	-0.106
28	4399	-0.092	3771	-0.791	4399	-0.045	4099	-0.438
29	4697	0.126	3810	-0.131	4438	-0.057	4219	1.097
30	4847	0.601	3997	-0.2	4697	0.099	4355	-0.085
31	4951	0.019	4054	-0.119	4847	0.298	4890	0.071
32	5039	0.083	4099	-0.087	4951	0.019	4927	-0.16
33	5230	-0.024	4219	1.208	5039	0.08	4957	0.041
34	5361	-0.169	4263	-0.103	5361	-0.365	5077	-0.074
35	5376	0.034	4355	-0.186	5466	-0.643	5145	-1.21
36	5466	-0.73	4504	-0.028	5833	0.054	5323	-0.015
37	5657	-0.034	4553	-0.178	6049	-0.278	5372	-0.031
38	6041	0.082	4927	-0.16	6162	0.054	5680	0.159
39	6049	-0.282	4957	0.07	6308	0.019	5726	0.662
40	6162	0.055	5077	-0.235	6477	-0.092	5781	0.202
41	6169	0.04	5145	-1.223	6854	-0.878	6067	-0.158
42	6308	0.034	5323	-0.406	6886	0.391	6258	-0.158
43	6477	-0.12	5372	-0.097			6383	0.029
44	6854	-0.822	5680	0.159			6422	-0.48
45	6886	0.495	5726	1.119			6541	-0.202
46			5781	0.443			6680	-0.904
47			5981	0.14			6712	-0.051
48			6067	-0.158			6839	0.696
49			6153	-0.021			6912	0.022
50			6258	-0.707			6929	-0.335
51			6422	-0.516			7105	-0.208
52			6680	-1.341				
53			6712	-0.124				
54			6839	0.996				
55			6886	0.107				
56			6912	0.029				
57			6929	-0.443				
58			7001	0.141				
59			7105	-0.345				

**Table 2:** Estimation results based on the probit model.

#	Setting 1		Setting 2		Setting 3		Setting 4	
	ID	EST	ID	EST	ID	EST	ID	EST
1	134	-0.103	7	0.081	412	-0.442	4	-1.633
2	263	0.020	53	0.015	490	-0.121	7	1.019
3	412	-0.101	183	-0.054	635	-0.389	83	0.137
4	738	0.035	268	-0.408	738	0.377	183	-0.120
5	758	-0.013	617	-0.040	760	0.271	225	0.054
6	760	0.433	727	-0.079	804	-1.178	731	-0.047
7	804	-0.432	731	-0.499	1144	-0.130	864	0.037
8	1144	-0.013	917	0.062	1207	-0.123	1108	1.407
9	1204	-0.053	1058	0.024	1239	-0.145	1148	1.343
10	1207	-0.065	1089	-0.048	1260	0.957	1307	0.233
11	1239	-0.072	1148	0.256	1685	-0.080	1357	-0.062
12	1260	0.168	1307	0.309	1834	1.297	1402	-0.028
13	1268	-0.022	1326	-0.053	1882	1.076	1687	-0.787
14	1381	-0.029	1565	0.047	1928	-0.980	1716	-0.038
15	1497	0.014	1588	-0.067	1997	0.069	1734	-0.056
16	1669	0.059	1687	-0.093	2288	1.473	1854	0.320
17	1685	-0.066	1708	-0.134	2354	-0.081	1858	0.031
18	1834	0.464	1858	0.424	2737	0.045	1906	-0.155
19	1882	0.433	1944	0.078	2797	0.042	1915	0.132
20	1928	-0.254	1987	0.327	2903	0.075	1987	0.103
21	1941	0.057	2039	0.375	3183	0.055	2039	0.918
22	2061	-0.069	2057	-0.098	3252	1.032	2161	-0.259
23	2288	0.460	2059	-0.026	3847	1.527	2240	-0.276
24	2426	0.052	2301	0.028	4211	-0.280	2373	0.194
25	2737	0.051	2377	0.696	4291	-0.987	2377	0.077
26	2797	0.090	2460	0.235	4438	-0.149	2443	-0.018
27	3144	-0.066	2534	-0.491	4697	0.125	2469	-0.070
28	3183	0.012	2682	0.070	4847	0.081	2661	-0.075
29	3252	0.465	2863	-0.077	5130	-0.115	2764	-0.030
30	3611	-0.016	2941	0.242	5140	-0.079	2809	0.078
31	3714	0.026	2984	-0.024	5361	-0.585	2941	0.050
32	3847	0.544	3016	0.031	5466	-0.598	3016	0.274
33	3932	-0.010	3205	-0.129	5833	0.215	3424	-0.093
34	4050	-0.068	3276	0.140	6049	-0.089	3446	0.051
35	4211	-0.138	3432	-0.026	6854	-1.044	3478	-0.145
36	4291	-0.152	3771	-0.229	6886	0.100	3493	-0.408
37	4399	-0.033	3810	-0.026			3494	0.084
38	4697	0.089	3814	0.067			3572	-0.163
39	4754	0.054	3997	-0.121			3697	-0.038
40	4847	0.130	4054	-0.177			3752	-0.030
41	4951	0.031	4219	0.477			3771	-0.114
42	4955	-0.011	4355	-0.074			3838	-0.032
43	5230	-0.016	4536	0.136			3997	-0.239
44	5290	-0.016	4600	-0.078			4099	-0.202
45	5361	-0.066	4927	-0.255			4103	-0.125
46	5376	0.087	4957	0.031			4219	0.631
47	5466	-0.224	5137	0.076			4458	-0.199
48	6162	0.033	5145	-0.578			4473	0.039
49	6169	0.065	5176	-0.042			4940	0.197
50	6308	0.068	5323	-0.119			5077	-0.339
51	6316	0.022	5372	-0.063			5249	-0.738
52	6854	-0.452	5379	-0.075			5303	0.123
53	6886	0.118	5551	0.013			5372	-0.785
54			5651	-0.033			5420	-0.030
55			5680	0.254			5484	-0.042
56			5726	0.454			5981	0.117
57			5781	0.317			6032	-0.066
58			5804	-0.013			6067	-0.157
59			5805	0.054			6153	-0.270
60			5881	-0.041			6180	0.241
61			5981	0.040			6326	1.097
62			6059	0.017			6341	0.031
63			6067	-0.251			6354	0.075
64			6258	-0.324			6383	0.382
65			6422	-0.273			6418	0.079
66			6451	-0.022			6521	0.156
67			6680	-0.673			6680	-0.148
68			6712	-0.082			6839	0.695
69			6839	0.498			6844	0.115
70			6886	0.136			6850	0.212
71			6929	-0.048			6885	-0.051
72			7001	0.116			6892	0.037
73			7090	0.078			6910	0.075
74			7105	-0.251			7090	0.080
75							7105	-1.395

**Table 3:** Summary of common genes based on the logistic regression model and the probit model.

#	Setting 1			Setting 2			Setting 3			Setting 4		
	ID	LR(1)	PM(2)	ID	LR(1)	PM(2)	ID	LR(1)	PM(2)	ID	LR(1)	PM(2)
1	134	-0.208	-0.103	7	0.410	0.081	412	-0.484	-0.442	731	-1.456	-0.047
2	412	-0.606	-0.101	268	-0.684	-0.408	635	-0.186	-0.389	1307	0.446	0.233
3	760	0.271	0.433	731	-1.161	-0.499	738	0.196	0.377	1687	-0.085	-0.787
4	804	-1.271	-0.432	1089	-0.133	-0.048	760	0.271	0.271	2469	-0.039	-0.070
5	1144	-0.090	-0.013	1148	0.475	0.256	804	-1.301	-1.178	4099	-0.438	-0.202
6	1207	-0.108	-0.065	1307	0.742	0.309	1144	-0.123	-0.130	5077	-0.074	-0.339
7	1239	-0.566	-0.072	1687	-0.153	-0.093	1207	-0.115	-0.123	6067	-0.158	-0.157
8	1260	0.786	0.168	1708	-0.229	-0.134	1239	-0.234	-0.145	6839	0.696	0.695
9	1669	0.155	0.059	1858	1.126	0.424	1260	0.912	0.957			
10	1685	-0.169	-0.066	1987	0.460	0.327	1834	1.496	1.297			
11	1834	1.471	0.464	2039	0.903	0.375	1882	1.271	1.076			
12	1882	1.337	0.433	2057	-0.175	-0.098	1928	-1.221	-0.980			
13	1928	-1.229	-0.254	2059	-0.169	-0.026	1997	0.155	0.069			
14	2288	0.414	0.460	2377	1.389	0.696	2288	0.858	1.473			
15	2737	0.051	0.051	2460	0.550	0.235	2354	-0.085	-0.081			
16	2797	0.052	0.090	2534	-0.969	-0.491	2737	0.054	0.045			
17	3183	0.376	0.012	2682	0.169	0.070	2797	0.075	0.042			
18	3252	0.464	0.465	2941	0.733	0.242	3252	0.801	1.032			
19	3714	0.078	0.026	3276	0.112	0.140	3847	1.553	1.527			
20	3847	1.529	0.544	3432	-0.378	-0.026	4211	-0.367	-0.280			
21	4211	-0.578	-0.138	3771	-0.791	-0.229	4291	-0.880	-0.987			
22	4291	-0.660	-0.152	3810	-0.131	-0.026	4438	-0.057	-0.149			
23	4399	-0.092	-0.033	3997	-0.200	-0.121	4697	0.099	0.125			
24	4697	0.126	0.089	4054	-0.119	-0.177	4847	0.298	0.081			
25	4847	0.601	0.130	4219	1.208	0.477	5361	-0.365	-0.585			
26	4951	0.019	0.031	4355	-0.186	-0.074	5466	-0.643	-0.598			
27	5230	-0.024	-0.016	4927	-0.160	-0.255	5833	0.054	0.215			
28	5361	-0.169	-0.066	4957	0.070	0.031	6049	-0.278	-0.089			
29	5376	0.034	0.087	5145	-1.223	-0.578	6854	-0.878	-1.044			
30	5466	-0.730	-0.224	5323	-0.406	-0.119						
31	6162	0.055	0.033	5372	-0.097	-0.063						
32	6169	0.040	0.065	5680	0.159	0.254						
33	6308	0.034	0.068	5726	1.119	0.454						
34	6854	-0.822	-0.452	5781	0.443	0.317						
35	6886	0.495	0.118	5981	0.140	0.040						
36				6067	-0.158	-0.251						
37				6258	-0.707	-0.324						
38				6422	-0.516	-0.273						
39				6680	-1.341	-0.673						
40				6712	-0.124	-0.082						
41				6839	0.996	0.498						
42				6886	0.107	0.136						
43				6929	-0.443	-0.048						
44				7001	0.141	0.116						
45				7105	-0.345	-0.251						
46												
47												
48												

We first examine Setting 1 where measurement error corrections are not incorporated. Based on BOOME, the logistic regression model retains 45 gene expression values, and the probit model suggests that 53 gene expression values should be included. Next, we explore the case that either the response or the predictors are corrected. Under Setting 2, the logistic regression model retains 59 gene expression values, and the probit model suggests that 74 gene expression values should be included. Under Setting 3, the logistic regression model retains 42 gene expression values, and the probit model suggests that 36 gene expression values should be included. Finally, under Setting 4 where measurement error effects response and the predictors are corrected, we have that the logistic regression model retains 51 gene expression values, and the probit model retains 75 gene expression values.

For the overall comparisons, we first observe that the variable selection result may depend on the correction of measurement error effects in the response and/or the predictors. The number of selected gene expressions under Setting 1 is almost smaller than that under other settings. For two regression models, the probit model retains more predictors than what the logistic regression model does, except for Setting 3. Finally, there are 35, 45, 29, and 8 gene expressions that are commonly selected by two models under Settings 1-4, respectively.

### 5.2 Demonstration of simulation studies

To show the validity of the BOOME method as well as the implementation of the package, we conduct simulation studies and demonstrate the programming code in this section.

Let *n* = 100 denote the sample size, and let *p* = 1000 or 5000 denote the dimension of predictors. For *i* = 1, …, *n*, we generate the *p*-dimensional vector of predictors *X*_*i*_ from the standard multivariate normal distribution. Let $\beta_{0}=(1,1,1,0_{p-3}^{⊤}{)}^{⊤}$ denote the true value of parameters, where 0_*q*_ represents the *q*-dimensional zero vector. Given *X*_*i*_ and *β*_0_, we generate the binary response *Y*_*i*_.

Noting that {(*Y*_*i*_, *X*_*i*_): *i* = 1, …, *n*} is regarded as *unobserved* data, we now generate error-prone data $\left\{(Y_{i}^{*},X_{i}^{*}):i=1,\ldots ,n\right\}$. For the generation of error-prone responses $Y_{i}^{*}$, we adopt the model (8), where misclassification probabilities *π*_*i*10_ and *π*_*i*01_ are formulated by logistic regression models. On the other hand, to generate error-prone predictors $X_{i}^{*}$, we adopt the model (7) with Σ_*ϵ*_ being specified as a diagonal matrix and diagonal entries are commonly specified as $\sigma_{\epsilon }^{2}=0.15,0.5$ or 0.75.

To see the data generation in details, we demonstrate the following code. We first specify the generation of predictors:

$$\begin{array}{ccc} & & \text{X}=[]\\ & & \text{for}\;\text{i}\;\text{in}\;\text{range}(1000):\\ & & \;\;\;\text{X}.\text{append}(\text{np}.\text{random}.\text{normal}(0,1,100))\\ & & \text{X}=\text{np}.\text{array}(\text{X}) \end{array}$$

Next, we specify the sample size and *β*_0_, and take *p* = 1000 and $\sigma_{\epsilon }^{2}=0.15$ as an example. Based on those information, we employ the function ME_Generate to generate error-pone data, where data represents the artificial data from the output of the function ME_Generate and pr represents two misclassification probabilities.

$$\begin{array}{ccc} & & \text{n}=100\\ & & \text{beta}=[1]*3+[0]*997\\ & & \text{cov}=\text{np}.\text{identity}(1000)*0.15\\ & & \text{gamma}=[[1],[0]*1000,[1],[0]*1000]\\ & & \text{ME}=\text{ME}\_\text{Generate}(\text{n},\text{beta},\text{cov},\text{X},\text{gamma})\\ & & \text{data}=\text{ME}[1]\\ & & \text{pr}=\text{ME}[0] \end{array}$$

Given the generated data, we define the response y and predictors x. To implement the BOOME method, we specify iteration number, values of *τ* and *η* to be ite = 1000, thres = 0.9, and lr = 0.00001, respectively. We now implement the function LR_Boost to examine the logistic regression model with measurement error in responses and predictors corrected. Detailed implementation and partial output are given below:

$$\begin{array}{ccc} & & \text{x}=\text{data}.\text{drop}(\left[\prime \text{y}\prime \right],\text{axis}=1)\\ & & \text{y}=\text{data}[\left[\prime \text{y}\prime \right]]\\ & & \text{ite}=1000\\ & & \text{thres}=0.9\\ & & \text{lr}=0.00001\\ & & \text{LR}\_\text{Boost}(\text{x},\text{y},\text{ite},\text{thres},1,1,\text{pr},\text{lr},\text{cov})\\ & & \text{estimated}\;\text{coefficient}:[1.02,1.04,0.99,0.0,0.0,0.0,0.0,0.0,0.0,\\ & & 0.0,0.0,0.0,0.0,0.0,0.0,0.0,0.0,0.0,0.0,0.0,0.0,0.0,0.0,0.0]\\ & & \text{predictors}:[1,2,3]\\ & & \text{number}\;\text{of}\;\text{predictors}:3 \end{array}$$

For the comparison with the proposed method with correction of measurement error in responses and predictors, we examine naive analysis based on Settings 1-3 in Section 5.1. Detailed implementation and partial outputs are given below. In general, we find from outputs that the first three estimator based on the proposed method is close to the true value 1, and selected predictors are the same as the underlying true setting. On the other hand, without correcting measurement error effects, we observe from the below results that the first three estimators have larger biases and are far from the true value 1. Moreover, additional irrelevant predictors are falsely included.

$$\begin{array}{ccc} & & \text{LR}\_\text{Boost}(\text{x},\text{y},\text{ite},\text{thres},0,0,\text{pr},\text{lr},\text{cov})\\ & & \text{estimated}\;\text{coefficient}:[-0.94,-1.13,-0.92,0.0,0.0,-0.20,0.0,-0.16,\\ & & 0.0,0.0,-0.11,0.0,0.0,0.14,0.0,0.0,0.0,0.0,0.0,0.0,0.0,0.0,\\ & & 0.0,0.0]\\ & & \text{predictors}:[1,2,3,6,8,11,14,129,148,229,300,346,374,421,436,\\ & & 453,471,480,498,520,523,543,562,589,590,628,631,634,639,640,\\ & & 650,668,684,704,768,774,798,851,936,965,983,985]\\ & & \text{number}\;\text{of}\;\text{predictors}:42 \end{array}$$

$$\begin{array}{ccc} & & \text{LR}\_\text{Boost}(\text{x},\text{y},\text{ite},\text{thres},1,0,\text{pr},\text{lr},\text{cov})\\ & & \text{estimated}\;\text{coefficient}:[-0.90,-1.20,-1.01,0.0,0.0,0.0,0.0,0.0,0.0,\\ & & 0.0,0.0,0.0,0.0,0.0,0.0,0.0,0.0,0.0,0.0,0.0,0.0,0.0,0.0,0.0]\\ & & \text{predictors}:[1,2,3,26,33,148,229,300,346,374,421,436,453,471,\\ & & 480,498,518,520,562,589,590,628,631,634,639,640,650,668,753,\\ & & 768,774,798,851,936,965,983,985]\\ & & \text{number}\;\text{of}\;\text{predictors}:37 \end{array}$$

$$\begin{array}{ccc} & & \text{LR}\_\text{Boost}(\text{x},\text{y},\text{ite},\text{thres},0,1,\text{pr},\text{lr},\text{cov})\\ & & \text{estimated}\;\text{coefficient}:[0.94,1.17,0.92,0.0,0.0,0.0,0.0,0.0,0.0,\\ & & 0.0,0.0,0.0,0.0,0.0,0.0,0.0,0.0,0.0,0.0,0.0,0.0,0.0,0.0,0.0]\\ & & \text{predictors}:[1,2,3,26,33,129,148,229,300,346,374,421,436,453,\\ & & 471,480,498,520,523,543,562,589,590,628,631,634,639,640,650,\\ & & 668,684,704,768,774,798,851,936,965,983,985]\\ & & \text{number}\;\text{of}\;\text{predictors}:40 \end{array}$$

In addition to the logistic regression models, we further examine the probit model based on four settings as described in Section 5.1. Specifically, we implement the function PM_Boost to construct the probit model and specify arguments (correct_X, correct_Y) to be (0,0), (0,1), (1,0), and (1,1) that reflect Settings 1-4 in Section 5.1, respectively. Detailed implementation and partial outputs are available below. Similar to the findings based on logistic regression models, we observe that the estimator with measurement error in responses and predictors corrected outperforms other scenarios because of smaller biases and precise variable selection. As expected, without suitable variable selection, the estimators induce tremendous biases and some irrelevant predictors are included.

$$\begin{array}{ccc} & & \text{PM}\_\text{Boost}(\text{x},\text{y},\text{ite},\text{thres},1,1,\text{pr},\text{lr},\text{cov})\\ & & \text{estimated}\;\text{coefficient}:[1.02,0.98,0.97,0.0,0.0,0.0,0.0,0.0,0.0,\\ & & 0.0,0.0,0.0,0.0,0.0,0.0,0.0,0.0,0.0,0.0,0.0,0.0,0.0,0.0,0.0]\\ & & \text{predictors}:[1,2,3]\\ & & \text{number}\;\text{of}\;\text{predictors}:3 \end{array}$$

$$\begin{array}{ccc} & & \text{PM}\_\text{Boost}(\text{x},\text{y},\text{ite},\text{thres},0,0,\text{pr},\text{lr},\text{cov})\\ & & \text{estimated}\;\text{coefficient}:[1.08,1.06,1.28,-0.02,-0.03,-0.01,0.0,-0.01,\\ & & -0.03,-0.02,0.0,0.10,0.0,-0.02,0.02,-0.045,0.04,0.0,0.06,-0.02,\\ & & 0.05,0.01,0.0,-0.01]\\ & & \text{predictors}:[1,2,3,4,5,6,8,9,10,12,14,15,16,17,19,20,21,22,\\ & & 24,25,26,27,29,30,31,32,33,34,35,37,38,39,40,41,43,44,45,\\ & & 47,48,49,50,51,52,53,54,55,56,57,59,60,62,63,65,66,67,68,\\ & & 69,72,73,75,76,78,79,80,81,82,83,84,85,86,87,88,89,90,91,\\ & & 92,93,95,96,97,99,100]\\ & & \text{number}\;\text{of}\;\text{predictors}:82 \end{array}$$

$$\begin{array}{ccc} & & \text{PM}\_\text{Boost}(\text{x},\text{y},\text{ite},\text{thres},1,0,\text{pr},\text{lr},\text{cov})\\ & & \text{estimated}\;\text{coefficient}:[1.09,1.07,1.04,-0.03,-0.03,0.0,0.0,0.0,\\ & & -0.04,-0.02,0.0,0.12,0.0,-0.02,0.02,-0.06,0.05,0.0,0.07,0.0,\\ & & 0.06,0.0,-0.01,-0.01]\\ & & \text{predictors}:[1,2,3,4,5,9,10,12,14,15,16,17,19,21,23,24,25,\\ & & 26,27,30,31,32,33,35,37,38,39,40,41,43,44,45,47,48,49,50,\\ & & 51,52,53,54,55,56,57,59,60,62,63,65,66,67,68,69,72,73,74,\\ & & 75,76,78,79,80,81,82,83,84,85,87,88,89,90,91,92,93,95,96,\\ & & 97,99,100]\\ & & \text{number}\;\text{of}\;\text{predictors}:77 \end{array}$$

$$\begin{array}{ccc} & & \text{PM}\_\text{Boost}(\text{x},\text{y},\text{ite},\text{thres},0,1,\text{pr},\text{lr},\text{cov})\\ & & \text{estimated}\;\text{coefficient}:[0.91,0.94,0.92,0.0,0.0,0.0,-0.02,0.01,0.0,\\ & & 0.03,0.0,0.04,0.0,0.0,0.0,0.0,0.0,0.0,0.0,0.0,0.0,0.0,\\ & & 0.0,0.0]\\ & & \text{predictors}:[1,2,3,7,8,10,12,30,31,34,35,38,43,54,62,80,\\ & & 92,97]\\ & & \text{number}\;\text{of}\;\text{predictors}:18 \end{array}$$

Finally, to precisely access the accuracy of the estimator, we use the *L*_1_-norm and the *L*_2_-norm, which are respectively defined as

$$\begin{array}{c} ∥\Delta_{\beta }{∥}_{1}=\sum_{i=1}^{p}\left|\hat{\beta_{i}}-\beta_{0,i}\right| \end{array}$$

and

$$\begin{array}{c} ∥\Delta_{\beta }{∥}_{2}=\sum_{i=1}^{p}{\left(\hat{\beta_{i}}-\beta_{0,i}\right)}^{2}, \end{array}$$

where $\Delta_{\beta }≜\hat{\beta }-\beta_{0}$, ${\hat{\beta }}_{i}$ and *β*_0,*i*_ are the *i*th entry of $\hat{\beta }$ and *β*_0_, respectively. To access the performance of variable selection, we examine specificity (SPE) and sensitivity (SEN), which are respectively defined as

$$\begin{array}{c} \text{SPE=}\frac{\text{the}\;\text{number}\;\text{of}\;\text{predictors}\;\text{that}\;\text{are}\;\text{correctly}\;\text{included}}{\text{the}\;\text{number}\;\text{of}\;\text{truly}\;\text{informative}\;\text{predictors}} \end{array}$$

and

$$\begin{array}{c} \text{SEN=}\frac{\text{the}\;\text{number}\;\text{of}\;\text{predictors}\;\text{that}\;\text{are}\;\text{correctly}\;\text{excluded}}{\text{the}\;\text{number}\;\text{of}\;\text{the}\;\text{truly}\;\text{unimportant}\;\text{predictors}}. \end{array}$$

Numerical results under all settings described above are reported in Table 4. We can observe that biases in the *L*_1_ and *L*_2_-norms are increasing when the magnitude of measurement error $\sigma_{\epsilon }^{2}$ and dimension *p* become large. As expected, when measurement error in responses and predictors are corrected (Setting 4), the biases are the smallest and SPE as well as SEN are the largest among all settings, which verify that the proposed method is valid to handle measurement error regardless of specification of regression models. On the other hand, without correcting measurement error effects, we find that the naive methods (Settings 1-3) produce significant biases and low values of SPE and SEN, indicating the worse performance of variable selection. In particular, if measurement error in responses and predictors are not corrected, as shown in Setting 1, we have the worst estimation results. Compared with Settings 2 and 3, it is interesting to see that the biases under Setting 2 are greater than those based on Setting 3, and values of SPE and SEN obtained by Setting 2 are smaller than those based on Setting 3. It implies that ignoring measurement error in predictors would incur severe biases and would be worse than ignoring measurement error effects occurred in responses.

**Table 4:** Simulation results for two regression models with *n* = 100.

Model	*p*	$\sigma_{\varepsilon }^{2}$	Setting	‖Δ_*β*_‖_1_	‖Δ_*β*_‖_2_	SPE	SEN
LR (1)	1000	0.15	1	4.633	1.807	0.900	0.213
			2	4.581	1.812	0.900	0.326
			3	0.358	0.184	1.000	0.945
			4	0.228	0.136	1.000	0.979
		0.50	1	4.400	1.780	0.667	0.230
			2	4.286	1.791	0.567	0.483
			3	1.758	0.606	1.000	0.583
			4	0.917	0.410	1.000	0.853
		0.75	1	4.244	1.772	0.767	0.249
			2	4.213	1.786	0.600	0.496
			3	2.458	0.789	1.000	0.417
			4	1.158	0.526	1.000	0.847
	5000	0.15	1	6.181	1.821	0.533	0.245
			2	5.794	1.818	0.467	0.400
			3	0.412	0.197	1.000	0.957
			4	0.256	0.147	1.000	0.981
		0.50	1	6.145	1.803	0.633	0.201
			2	5.485	1.802	0.233	0.458
			3	2.490	0.641	1.000	0.602
			4	1.082	0.427	1.000	0.889
		0.75	1	5.622	1.797	0.300	0.218
			2	5.122	1.806	0.533	0.529
			3	3.358	0.813	1.000	0.452
			4	1.445	0.581	1.000	0.862
PM (2)	1000	0.15	1	9.367	1.855	0.767	0.297
			2	7.890	1.829	0.567	0.459
			3	0.424	0.168	1.000	0.964
			4	0.234	0.124	1.000	0.988
		0.50	1	9.345	1.843	0.767	0.224
			2	7.168	1.821	0.267	0.545
			3	3.203	0.664	1.000	0.696
			4	1.598	0.507	1.000	0.887
		0.75	1	9.027	1.861	0.900	0.247
			2	7.105	1.860	0.567	0.555
			3	4.592	0.822	1.000	0.548
			4	2.301	0.654	1.000	0.837
	5000	0.15	1	16.555	1.994	0.400	0.452
			2	12.462	1.908	0.367	0.593
			3	0.419	0.145	1.000	0.979
			4	0.262	0.132	1.000	0.993
		0.50	1	17.391	2.031	0.667	0.233
			2	13.177	1.943	0.233	0.587
			3	1.244	0.204	1.000	0.819
			4	0.333	0.066	1.000	0.930
		0.75	1	17.903	2.043	0.633	0.419
			2	13.289	1.948	0.500	0.574
			3	1.445	0.194	1.000	0.738
			4	0.707	0.098	1.000	0.789

## 6 Discussion

In this paper, we introduce the Python package BOOME that aims to address ultrahigh-dimensional data subject to measurement error in responses and predictors. Unlike existing packages that deal with either variable selection or measurement error but not both, our package can handle variable selection and correct measurement error effects to both responses and predictors simultaneously. In addition, the computational time is fairly fast and arguments are flexible for public use. In applications, sometimes variables in datasets can be shown to be free of measurement error and can be precisely measured, such as age or gender. The package BOOME is still flexible to handle those scenarios. For example, if researchers believe that predictors in their datasets are free of measurement error, then they can adopt Setting 2 in Section 5.1 by employing corrected responses and precisely measured predictors; if only predictors are shown to have measurement error, then one can adopt Setting 3 in Section 5.1 by implementing corrected predictors and precisely measured responses.

There are several possible extensions based on the current developments. First, in addition to continuous or binary random variables, categorical or counted data are frequently adopted in the framework of bioinformatics, such as RNA sequence or GWAS data, and they might be subject to mismeasurement error. Therefore, it is important to propose a valid approach to adjust for measurement error effects to those data. In addition, our current approach focuses on parametric logistic regression or probit models. To provide general formulations, it is interesting to extend the BOOME method to nonparametric models or semi-pamatric models. In the current development, our attention primarily focuses on variable selection for high-dimensional data subject to measurement error. In supervising learning, examining the performance of classification and prediction is a crucial concern. Provided that additional information, such as validation samples, is available, it is interesting to adopt selected predictors and adjustments of measurement error from the BOOME method to define a general model of measurement heterogeneity and develop several measures (e.g., C-statistic or Brier score) to assess the predictive performance (e.g., [30]). In addition, since responses are subject to measurement error as well, it deserves careful exploration to handle measurement error in responses when doing prediction.

Finally, as commented by a referee, dimension reduction techniques, such as principal component analysis (PCA) or factor analysis, can be valid tools to reduce dimension from ultrahigh-dimensional predictors. However, there are two main issues in the current development. First, the purpose in this study is to detect informative predictors and exclude irrelevant ones, while dimension reduction techniques aim to reduce dimension through linear combinations of high-dimensional predictors. Second, when the predictors are subject to measurement error, the BOOME package is able to address measurement error effects and correctly retain important predictors, while correction of measurement error effects for dimension reduction techniques is not explored, especially when the response is contaminated with measurement error as well. Undoubtedly, it is an interesting perspective to handle ultrahigh-dimensional data and deserves careful exploration in the future research.
